# Microstructural changes in the reward system are associated with post-stroke depression

**DOI:** 10.1016/j.nicl.2020.102360

**Published:** 2020-07-22

**Authors:** Lena K.L. Oestreich, Paul Wright, Michael J. O'Sullivan

**Affiliations:** a UQ Centre for Clinical Research, The University of Queensland, Brisbane, Australia; b Centre for Advanced Imaging, The University of Queensland, Brisbane, Australia; c Department of Neuroimaging, Institute of Psychiatry, Psychology and Neuroscience, King’s College London, UK; d Department of Neurology, Royal Brisbane and Women’s Hospital, Brisbane, Australia; e Herston Imaging Research Facility, Royal Brisbane and Women’s Hospital, Brisbane, Australia

**Keywords:** Post-stroke depression (PSD), Magnetic resonance imaging (MRI), Neuroinflammation, White matter, Grey matter, Connectome

## Abstract

**Background:**

Studies of lesion location have been unsuccessful in identifying mappings between single brain regions and post-stroke depression (PSD). Based on studies implicating the reward system in major depressive disorder without stroke, we investigated structural correlates within this system and their associations with PSD.

**Methods:**

The study enrolled 16 healthy controls, 12 stroke patients with PSD and 34 stroke patients free of PSD. Participants underwent 3T structural and diffusion MRI. Graph theoretical measures were used to examine global topology and whole-brain connectome analyses were employed to assess differences in the interregional connectivity matrix between groups. Structural correlates specific to the reward system were examined from grey matter volumes and by reconstructing its main white matter pathways, namely the medial forebrain bundle and cingulum connections, using deterministic tractography. Fractional anisotropy (FA) was derived as a measure of microstructural organization, and extracellular free-water (FW) as a possible proxy of neuroinflammation.

**Results:**

Subnetworks of decreased FA-weighted and increased FW-weighted connectivity were observed in patients with PSD relative to healthy controls. These networks subsumed the majority of regions constituting the reward system. Within the reward system, FA and FW of major connection pathways and grey matter volume were collectively predictive of PSD, explaining 37.8% of the variance in depression severity.

**Conclusions:**

PSD is associated with grey matter volume loss, reduced FA and increased extracellular FW in the reward system, similar to features observed in major depression without stroke. Structural characterization of the reward system is a promising biomarker of vulnerability to depression after stroke.

## Highlights

• Depressed stroke survivors exhibit abnormal frontal and subcortical structural connectivity.
• Microstructural changes resemble those reported in major depressive disorder.
• Subnetworks linked with reward processing are associated with poststroke depression.
• Localized tractography confirmed involvement of connections in these networks.
• Grey matter volume, fractional anisotropy and free-water collectively predict depression severity.

## 1 Introduction

Post-stroke depression (PSD) is a common complication after stroke, with approximately 31% of stroke survivors meeting the criteria for major depression 3–6 months after stroke (Hackett and Pickles, 2014). Patients with PSD have increased disability, mortality and poorer rehabilitation outcomes, compared with individuals free of depression (Paolucci, 2008). Despite these well-known, detrimental effects on functional recovery, recognition and treatment of PSD remains suboptimal (Paolucci, 2008). A possible factor is a poor understanding of the underlying brain-based biological mechanisms. Studies that have investigated associations between lesion location and PSD have generated inconsistent and often contradictory findings, leaving the field unable to reach a consensus for the mechanistic basis of PSD (Wei et al., 2015). Early qualitative approaches based on visual inspection to determine lesion location reported higher incidences of PSD in patients with left hemisphere lesions (Robinson et al., 1984, Robinson and Price, 1982), but were soon complemented by studies showing the opposite pattern, with an association between PSD and right hemisphere lesions (MacHale et al., 1998, Rosse and Ciolino, 1985). More recent studies using voxel-based lesion symptom mapping, which normalizes and co-registers brain imaging data into a standard template and therefore represents a more quantitative approach to study lesion locations, have also reported conflicting findings (Gozzi et al., 2014, Kim et al., 2017, Terroni et al., 2011). This inconsistency among single studies is reflected in systematic reviews (Carson et al., 2000, Nickel and Thomalla, 2017, Wei et al., 2015) and meta-analyses (Narushima et al., 2003, Yu et al., 2004), which have been unable to reveal any straightforward association between lesion location and PSD.

The inability to pinpoint lesion locations specific to PSD has led some to question whether PSD might arise from more diffusely distributed pathogenic mechanisms, such as widespread activation of inflammatory mechanisms. Systemic inflammation has recurrently been implicated in major depressive disorder (MDD) (Bullmore, 2018). However, accumulating evidence suggests that even in the presence of systemic inflammation, the causative alterations reside in relatively circumscribed brain regions (Russo and Nestler, 2013). Structural connectome studies reported disrupted white matter connectivity (Korgaonkar et al., 2014, Singh et al., 2013) primarily localized to subcortical-frontal regions in MDD. Furthermore, a connectome study in PSD reported impaired network integration and segregation in fronto-limbic regions to be associated with depression severity (Xu et al., 2019). These brain circuits are commonly referred to as the reward system, which is essential for emotional and motivational information processing and plays a pivotal role in memory (Russo and Nestler, 2013). It consists of subcortical and fronto-cortical regions (Russo and Nestler, 2013), which are interconnected by white matter projections of the cingulum bundle and medial forebrain bundle. Multiple neuroimaging studies have provided evidence for grey matter volume reductions in the reward system (Enneking et al., 2019, Sin et al., 2018) and microstructural changes in the medial forebrain bundle (Bracht et al., 2015, Jia et al., 2014) and the cingulum bundle (Bracht et al., 2015, de Diego-Adelino et al., 2014) to be implicated in MDD.

Based on the lack of consistent lesion locations associated with PSD, together with mounting evidence for structural changes in the reward system associated with MDD, we set out to investigate network level and reward system structural features as substrates of PSD. Structural connectome analyses and global topology were used to assess connectivity differences between stroke patients with and without PSD, and healthy controls. We then investigated structural changes specifically localized to the reward system by reconstructing its main white matter pathways and parcellating its main grey matter structures. We hypothesized that patients with PSD would exhibit connectivity disruptions relative to healthy controls and stroke patients without PSD particularly in structures constituting the reward circuit. We furthermore hypothesized that depressive symptom severity would be associated with microstructural alterations in patients with recent stroke.

## 2 Materials and methods

### 2.1 Participants

Patients with first ischemic stroke were enrolled into a longitudinal study (STRATEGIC) within 7 days of stroke. Inclusion criteria were age over 50 years and clinical stroke confirmed by CT or MRI. Exclusion criteria were dementia, previous stroke, inability to converse fluently in English, major neurological disease, active malignancy, previous moderate to severe head injury and any other factor that would prevent performance of cognitive tasks (e.g. visual impairment). The study procedures were approved by the London and Bromley Research Ethics Committee and the University of Queensland Research Ethics Committee. All participants gave written informed consent. Out of 179 stroke patients enrolled in the STRATEGIC study, a sample of 46 stroke patients were included in this study. This subset of participants was recruited for research MRI and more detailed cognitive and behavioral evaluation. This evaluation included the Geriatric Depression Scale (GDS), a 30-item self-report measure to identify depression in older people (Yesavage et al., 1982) performed 27–82 days after stroke (*M* = 42.95, *SD* = 13.95) and MRI 30–95 days after stroke (*M* = 65.76, *SD* = 17.16). In addition to the 46 patients, 16 healthy controls were recruited from the community (see Table 1). Participants (*n* = 62) ranged in age from 51 to 86 years (*M* = 70.04, *SD* = 9.07), 37.1% (*n* = 23) were female, and 97% (*n* = 60) were right-handed (see Table 1).

**Table 1:** Demographics, risk factors and lesion characteristics by group.

	HC (*n* = 16)	D+ (*n* = 12)	D− (*n* = 34)	
Variable	Mean (*SD*)/category	Mean (*SD*)/category	Mean (*SD*)/category	Group comparisons
*Demographics*				
Age (years)	71.53 (10.62)	69.72 (6.96)	69.46 (9.13)	*F*(2,59) = 0.29, *p* = 0.75
Sex (female/male)	11/5	3/9	9/25	*χ^2^*(2) = 9.27, *p* = 0.01
Handedness (right/left)	16/0	11/1	33/1	*χ^2^*(2) = 1.55, *p* = 0.47
*Vascular risk factors*				
ECG (normal/sinus rhythm/atrial fibrillation)	n/a	1/10/1	1/23/10	*χ^2^*(2) = 2.55, *p* = 0.28
Hypertension (no/yes^a^/yes^b^)	n/a	5/7/0	15/15/4	*χ^2^*(2) = 1.8, *p* = 0.41
Diabetes mellitus (no/yes^c^/yes^d^/yes^e^/yes^f^)	n/a	10/0/1/1	28/3/3/0	*χ^2^*(3) = 3.9, *p* = 0.27
Smoking (never/previously/current)	n/a	5/6/1	19/10/5	*χ^2^*(2) = 1.7, *p* = 0.43
Ischemic heart disease (no/yes)	n/a	10/2	27/7	*χ^2^*(1) = 0.09, *p* = 0.77
Statins (no/yes^g^/yes^h^)	n/a	1/7/4	2/21/11	*χ^2^*(2) = 0.1, *p* = 0.95
*Vascular disease*				
Small vessel disease (no/yes)	n/a	7/5	22/12	*χ^2^*(1) = 0.16, *p* = 0.69
WMH (Fazekas: 0/1/2/3)^i^	n/a	0/6/5/1	0/14/13/7	*χ^2^*(3) = 0.95, *p* = 0.62
*Lesion characteristics*				
Hemisphere (left/right)		5/7	19/15	*χ^2^*(1) = 0.72, *p* = 0.4
Arterial territory (MCA_ant_/MCA_pos_/MCA_str_/PCA/lacunar/thalamic)		2/3/3/2/1/1	7/7/5/8/5/2	*χ^2^*(5) = 1.24, *p* = 0.94
Volume (ml)		7175.64 (12081.03)	7947.09 (11917.81)	*t*(44) = 0.73, *p* = 0.47
Time since lesion (days)		68.17 (13.39)	79.88 (54.63)	*t*(44) = 0.19, *p* = 0.85

*Note.* HC = healthy controls; D+ = Depression group; D− = no Depression group; SD = standard deviation; ECG = electrocardiogram; ^a^controlled, ^b^uncontrolled; ^c^type 1, ^d^type 2 controlled by diet, ^e^type 2 controlled by tablets, ^f^type 2 controlled by insulin injections; ^g^normal lipids, ^h^abnormal lipids; WMH = white matter hyperintensities, DWM = deep white matter, ^i^Fazekas scores: periventricular white matter: 0 = absent, 1 = “caps” or pencil-thin lining, 2 = smooth “halo”, 3 = irregular periventricular signal extending into deep white matter, deep white matter: 0 = absent, 1 = punctate foci, 2 = beginning confluence, 3 = large confluent areas; MCA_ant_ = middle cerebral artery, anterior; MCA_pos_ = middle cerebral artery, posterior; MCA_str_ = middle cerebral artery, striatocapsular; PCA = posterior cerebral artery

The GDS has been reported to have an excellent internal consistency (Cronbach’s alpha = 0.89) (Sivrioglu et al., 2009) and excellent concurrent validity with the Hamilton Rating scale (r = 0.82; (Agrell and Dehlin, 1989) in older adults and stroke patients. A cutoff score of 10 on the GDS has previously been identified to yield the highest sensitivity (0.69) and specificity (0.75) in patients with PSD (Sivrioglu et al., 2009). Thirty-four (73.9%) stroke patients had a score below 10 on the GDS and were assigned to the group without PSD (D-). The remaining 12 (26.1%) participants scored 10 or above on the GDS and were assigned to the PSD group (D+).

### 2.2 Data acquisition

MRI scans were collected on a 3T MR750 MR scanner (GE Healthcare, Little Chalfont, Buckinghamshire, UK). T1-weighted images were acquired with the MPRAGE sequence (Marques et al., 2010) with a repetition time (TR) of 7.312 ms, echo time (TE) of 3.016 ms and a flip angle of 11°. Images were acquired in the sagittal plane with field of view (FOV) of 270 × 270 mm, matrix size of 256 × 256 voxels and slice thickness and gap of 1.2 mm. T2-weighted fluid-attenuated inversion recovery (FLAIR) and fast recovery fast spin echo (FRFSE) sequences were acquired for infarct and lesion delineation. The FLAIR sequence was acquired with TR of 8000 ms, TE of 120–130 ms and flip angle of 90-111°. The FRFSE sequence used TR of 4380 ms, TE of 54–65 ms and flip angle of 90-111°. Images were acquired in the axial plane with FOV of 240 × 240 mm for both sequences. The matrix size for the FLAIR sequence was 256 × 128 voxels and 320 × 256 voxels for the FRFSE sequence. Slice positions were aligned for both sequences with 36 slices at 4 mm thickness for FLAIR and 72 slices at 2 mm thickness for FRFSE.

Diffusion-weighted images were acquired with an echo planar imaging sequence with double refocused spin echo for 60 diffusion-sensitization directions at *b* = 1500 s/mm^2^ and six acquisitions without diffusion sensitization (*b* = 0). Image geometry for the diffusion-weighted images covered the whole brain using 2 mm axial slices with matrix size of 128 × 128 voxels and FOV of 256 × 256 mm, resulting in 2 mm isotropic resolution. Participants’ heads were aligned such that the intercommissural line was as close to the axial plane as possible. Acquisition was peripherally gated to the cardiac cycle, giving a sequence duration of 11–20 min, a TR of 10,000–14,118 ms and a TE of 66–78 ms with a flip angle of 90°.

### 2.3 Lesion definition

Lesions were drawn manually on FLAIR images. When necessary, diffusion images acquired acutely were used to identify the infarct. Lesion volume was calculated from FLAIR images. Images containing lesion maps were co-registered into Montreal Neurological Institute (MNI) standard space, so that anatomically homologous brain areas were aligned.

### 2.4 Connectome reconstruction

Cortical and subcortical parcellations were reconstructed from the T1-weighted images using the *recon-all* command implemented in FreeSurfer (v6.0) (http://surfer.nmr.mgh.harvard.edu/) and validated by manual inspection. Parcellations for the connectome reconstruction were based on the Desikan-Killiany atlas, resulting in 84 connectome nodes (Desikan et al., 2006). The diffusion-weighted data were pre-processed using tools implemented in MRtrix3 (Tournier et al., 2012) to correct for head movements, eddy current distortions and magnetic field inhomogeneities (Tournier et al., 2012). A five-tissue-type segmented image was generated from the pre-processed T1-weighted images. Response functions were estimated using the single-fibre *tournier* algorithm (Tournier et al., 2013) and constrained spherical deconvolution was applied to obtain fibre orientation distributions (FOD). Anatomically constrained tractography with the 2nd order integration over Fibre Orientation Distribution (iFOD2) algorithm (Tournier et al., 2012), was used to generate individual tractograms for each participant (Smith et al., 2012). Tractograms were generated until 100 million streamlines were obtained with a length of 5–250 mm, step size of 1 mm and FOD amplitude threshold of 0.1. The spherical-deconvolution informed filtering of tractograms (SIFT) algorithm was applied to reduce the overall streamline count to 10 million streamlines, providing more biologically meaningful estimates (Smith et al., 2013). Individual connectivity matrices were manually inspected for missing connections (edges) to nodes located in lesions. Free-water imaging was used on the pre-processed DWI data to quantify the amount of extracellular free-water (FW) by separating the diffusion properties of brain tissue, such as white matter tracts, from the surrounding extracellular free water, such as cerebrospinal fluid (Pasternak et al., 2009). Partial volume effects were removed before estimating fractional anisotropy (FA; Pasternak et al., 2009). Individual tractograms were reconstructed and connectivity matrices were generated by mapping streamlines onto nodes of each participant’s parcellation image. Separate connectivity matrices were populated with FA and FW.

### 2.5 Graph theoretical measures

In order to investigate whether microstructural changes would impact global brain connectivity, whole-brain structural graph theoretical analyses were conducted on the interregional connectivity matrices using the Brain Connectivity Toolbox (http://www.brain-connectivity-toolbox.net). A sparsity threshold was applied to facilitate global network comparisons. This ensures that the number of edges are matched across participants by retaining 90% of the top connections for each participant. The sparsity threshold facilitates the exclusion of connections with connectivity strengths of 0 across all subjects from hypothesis testing and before calculation of global network measures. Global efficiency, modularity and global clustering coefficient were calculated. These graph measures were chosen because they have been well studied in major depressive disorder (Gong and He, 2015) and their interpretations are generally well accepted (Rubinov and Sporns, 2010). Global efficiency estimates the brain’s capacity for parallel information transfer and overall integration of a network. As such, it may give an indication as to whether structural connectivity across disparate regions of the brain is preserved in PSD. Modularity and average global clustering coefficient were calculated as measures of brain segregation: modularity estimates the density of interconnected clusters, which have few sparse connections to nodes from other clusters and therefore represents a metric of a network’s segregation into multiple subnetworks. Modularity was calculated with Neman’s spectral community detection, implemented in the Brain Connectivity Toolbox. Average global clustering coefficient, which quantifies the connectivity strength of all closed triangles a node forms with its neighbouring nodes, provides an estimate of the extent to which the neighbours of a node can build a complete graph. Measures of brain segregation may provide information of potential topological reorganization as a consequence of stroke which may be associated with PSD.

### 2.6 Measurements from the reward system

Grey matter regions of the reward system were selected based on a literature review of fMRI and structural MRI studies of the reward system in depression (see Table S1). The identified regions included seven subcortical structures, i.e. amygdala (Amy), nucleus accumbens (NAc), thalamus (Th), hippocampus (HPC), caudate (Cau), putamen (Pu) and ventral tegmental area (VTA) and five cortical structures, i.e. dorsolateral prefrontal cortex (dlPFC), medial prefrontal cortex (mPFC), orbitofrontal cortex (OFC), anterior cingulate cortex (ACC) and insula (Ins). Grey matter volumes were calculated for all identified regions constituting the reward system, except the VTA. The VTA is a very small area, which has not yet been well characterized structurally or functionally in human MRI studies (Russo and Nestler, 2013) and was therefore excluded. The remaining grey matter regions were defined by the cortical and subcortical FreeSurfer parcellations (http://surfer.nmr.mgh.harvard.edu/) derived from the segmentation of the T1-weighted images based on the Desikan-Killiany atlas also used for the connectome analysis.

**Fig. 1:**
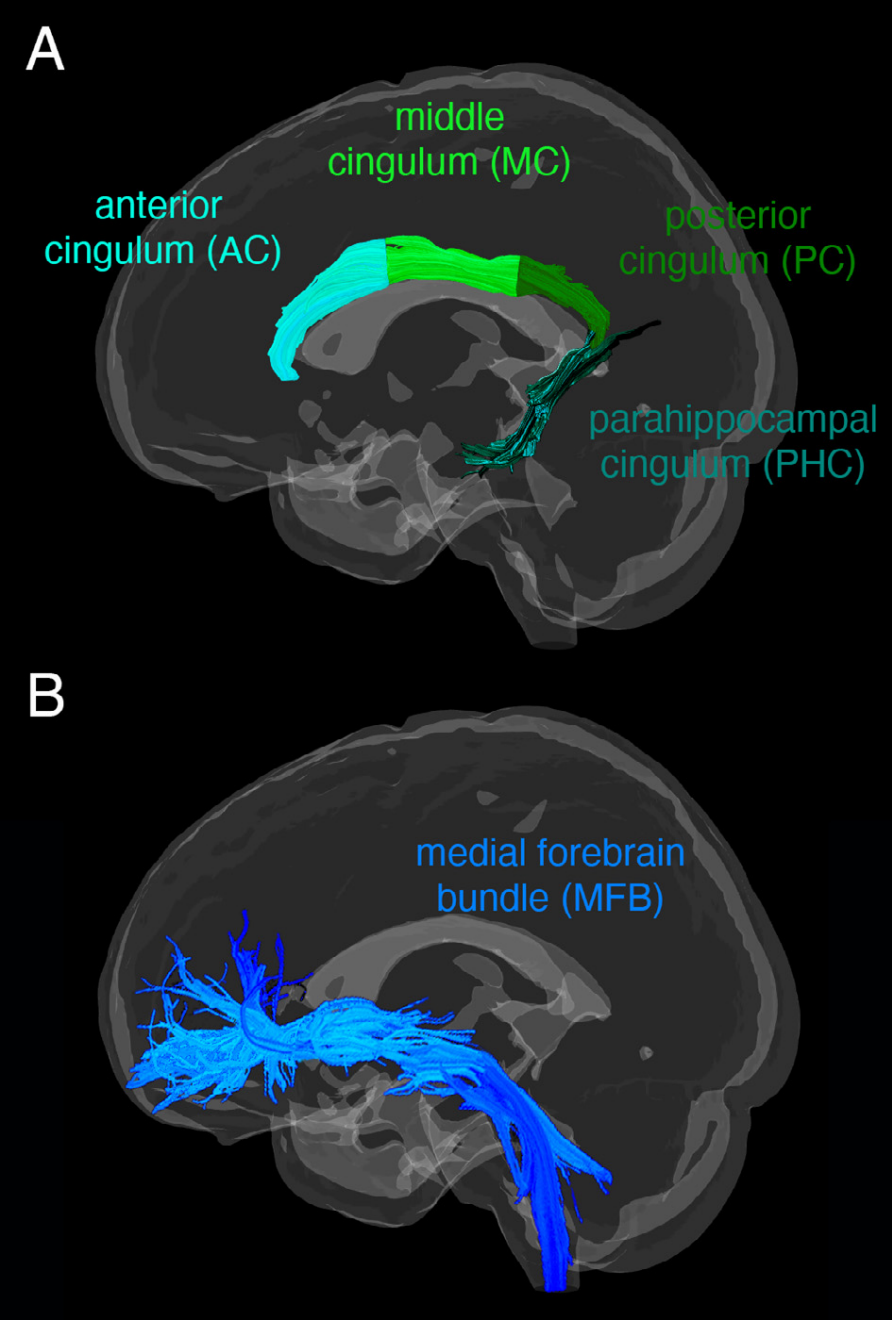
A) Cingulum bundle subdivisions and B) Medial forebrain bundle (MFB) reconstructed with anatomically constrained tractography from one representative participant.

The cingulum bundle and medial forebrain bundle (MFB), were reconstructed with anatomically constrained tractography in MRtriX3 (Tournier et al., 2012) using a deterministic tractography algorithm (see Fig. 1). The cingulum bundle was divided into anterior, middle, posterior and parahippocampal subdivisions and the MFB was reconstructed as a single tract in each hemisphere. A detailed description of the protocols used for the tractography procedure is provided in the supplements. The deterministic tractography algorithm based on spherical deconvolution, which was used for the reconstruction of the cingulum subdivisions and MFB takes the FOD image as input and samples it at each streamline step. The Newton optimization is performed on the sphere from the current streamline tangent orientation to locate the orientation of the nearest FOD amplitude peak. The step size of the tracking algorithm was set to 0.5 mm, with a cut-off value for the FOD amplitude of 0.05 and maximum turning angle of 45°. The minimum pathlength for the cingulum subdivisions was 10 mm and for the MFB 30 mm. Reproducibility of the cingulum subdivisions and MFB were assessed with the inter-rater reliability between two raters on a subset of 10 randomly chosen participants. The ICC ranged from 0.89 to 0.97 for FA and 0.85–0.98 for FW, indicating that the interrater reproducibility was very good for all tract segments. FA and FW were averaged across each tract.

### 2.7 Statistical analysis

Sex was added as a covariate to all analyses. Voxel-based symptom lesion mapping (VSLM) was performed on the co-registered lesion images using the toolbox NiiStat (NiiStat, RRID:SCR_014152) implemented in MATLAB 2018b (“MATLAB and Statistics Toolbox,” 2018). Due to the fact that previous VLSM studies were unable to identify associations between lesion locations and PSD and the relatively small sample size of this study, statistical maps of association are presented unthresholded and report the maximum effect sizes in this sample, so that even weak effects may be detected. A univariate linear regression analysis was conducted with lesion location as independent variable and GDS scores (depression severity) as outcome variable, using 5000 permutations.

In order to test for whole-brain topological differences between groups, a multivariate analysis of covariance (MANCOVA) was performed with the independent variable *group* (HC/D−/D+) and the six dependent variables *global efficiency (FA/FW), modularity (FA/FW),* and *centrality coefficient (FA/FW),* using SPSS 25.0 (Corp., 2017). Post-hoc comparisons were adjusted for multiple comparisons using the Bonferroni correction at an alpha level of 0.05. Network-based statistics (NBS, https://sites.google.com/site/bctnet/comparison/nbs) were used to investigate whole-brain between-group (HC/D−/D+) differences in FA and FW (Zalesky et al., 2010). Supra-threshold connections were considered if their test-statistic exceeded a *p*-value of <0.001 with 10,000 permutations (*t*-statistic >3). More conservative test-statistic supra-thresholds of 3.5 (*p* < 0.0005) and 4 (*p* < 0.0001) were also tested. Subnetworks (connected components) were then identified with a family-wise error (FWE)-corrected *p*-value of 0.05.

All remaining statistical analyses were performed using SPSS 25.0 (Corp., 2017). To test for structural group differences in the reward system, three repeated-measures analysis of covariance (ANCOVAs) were performed with *group* (HC/D−/D+) as between- subjects factor and *hemisphere* (left/right) as within-subjects factor. Each of the ANCOVAs included an additional within-subjects factor, i.e. *FA* (anterior cingulum/middle cingulum/posterior cingulum/parahippocampal cingulum/MFB), *FW* (anterior cingulum/middle cingulum/posterior cingulum/parahippocampal cingulum/MFB) or *grey matter volume* (HPC/Th/NAc/Amy/Cau/Pu/Ins/dlPFC/mPFC/OFC/ACC), respectively. Post-hoc comparisons were adjusted for multiple comparisons using the Bonferroni correction at an alpha level of 0.05. A final ANCOVA was performed in stroke patients only, to test for possible associations between vascular risk factors of stroke (*ECG*/*hypertension*/*diabetes mellitus*/*smoking*/*ischemic heart disease*/*statins,* see Table 1) and underlying vascular disease (*small vessel disease*/*white matter hyperintensities*) with measures from the reward system.

In order to investigate whether global graph theoretical metrics or structural changes in the reward system are significantly associated with PSD severity, two multiple linear regression analysis were performed in the stroke sample only. All independent variables and covariates were demeaned. Sex, age and handedness are known to vary with brain structure and the role of lesion characteristics (lesion volume, lesion hemisphere, lesion location by arterial territory and days since lesion) on depression severity is still unclear. These variables were therefore added as covariates. Covariates that correlated significantly or at a trend-level (*p* < 0.1) with GDS scores were included in the final models. For this purpose, two-sided Pearson correlations were performed between the covariates and GDS scores. Only handedness (*r* = 0.336, *p* = 0.022) and days since lesion (*r* = −0.404, *p* = 0.01) were correlated with GDS scores and were added as covariates in the final regression analyses. In the first multiple linear regression analysis, all global network measures were added as predictors and GDS scores were entered as outcome variable. The second regression was run as a stepwise multiple linear regression analysis, to limit the number of predictors in the model and thereby reduce the chance of inflation of results. To further minimize the risk of model overfitting, we employed a cross-validation approach, whereby the data was randomly split into two samples. The first sample consisted of 70% of participant (training set), which was used to estimate the regression model. The model was then tested on the remaining 30% of participants (test set). GDS scores were again entered as outcome variable. Variance inflation factors (VIF) were calculated to check for multicollinearity. Cutoff values for VIF were set to 5 and effects with VIFs > 5 were deemed too large to keep in the model (Craney and Surles, 2002).

One participant had a GDS score of more than 3 times the standard deviation from the sample mean. Due to the fact that this score is a possible and valid score on the GDS, we decided not to remove the participant all together but instead conducted the regression analysis once with and once without this data point. Outliers of the graph measures and measures of the reward system were defined as 1.5 times the interquartile (IQ) range and were assessed for all variables and analyses.

## 3 Results

No statistical outliers were detected on any metrics in the dataset. The three groups did not differ significantly on age or handedness, but there were more women among the healthy controls and more men in the stroke groups but a similar gender ratio (approximately 1:3 women to men) in both D+ and D− groups (*χ^2^*(1) = 0.01, *p* = 0.921, see Table 1). While data on vascular risk factors or vascular disease were not available for the HC group, the D+ and D− groups did not differ significantly on any measures of vascular risk factors, vascular disease or lesion characteristics (see Table1).

### 3.1 Associations between depression and lesion characteristics

**Fig. 2:**
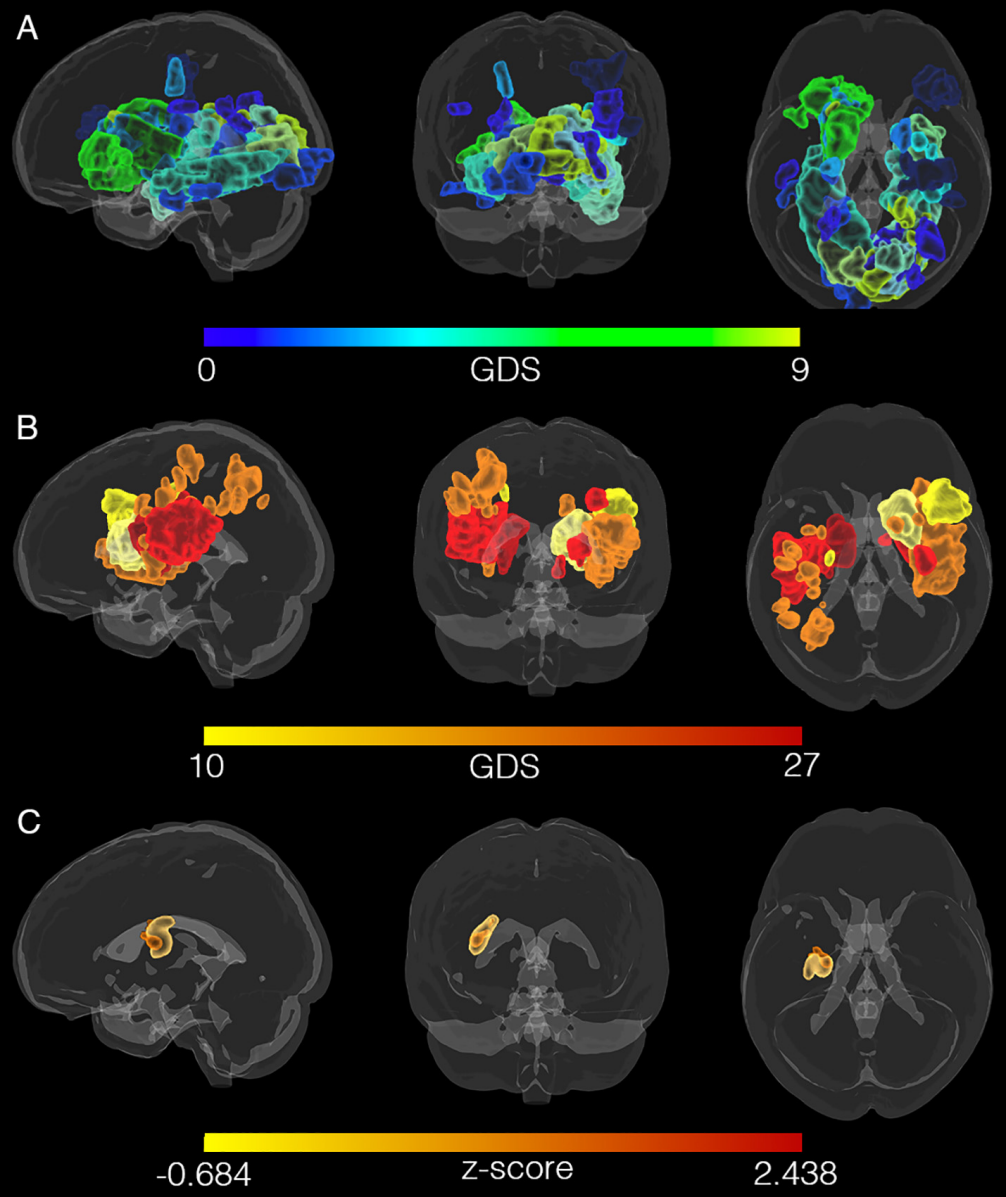
Lesion locations mapped for all participants in standard (MNI) space does not reveal a pattern of association between GDS scores and lesion location. A) Lesions mapped for participants without depression, i.e. GDS scores from 0 (green) to 9 (blue). B) Lesions mapped for participants with depression, i.e. GDS scores from 10 (yellow) to 27 (red). C) VLSM maps calculated for GDS scores of 46 stroke patients. The (uncorrected) cluster includes areas of the medial forebrain bundle (MFB) and the putamen in the left hemisphere. (For interpretation of the references to color in this figure legend, the reader is referred to the web version of this article.)

There was no simple relationship between lesion location and the presence of depression, apparent on visual inspection of the lesion overlay maps of the D+ and D− groups (see Fig. 2). VLSM identified a small cluster of 1195 voxels in the left putamen and part of the MFB (see Fig. 2C) associated with GDS scores but the strength of association was modest (*z* = 2.438, *p_uncorr_* = 0.008). No significant clusters were obtained when correcting the p-value for multiple comparisons.

### 3.2 Whole-brain topology and connectome analysis

One participant had no connections (edges) to the postcentral gyrus due to a lesion in this area. Connectivity matrices for all other participants were fully connected, i.e. all nodes were connected to other nodes. There was a significant main effect of *group* on *modularity FW* (*F*(3,61) = 2.239, *p* = 0.046, *η_p_^2^* = 0.101). Polynomial contrasts of *group* identified a significant linear increase in *modularity FW* from the HC group, to the D− and the D+ group (*t*(59) = 2.394, *p* = 0.02). The D+ group had significantly increased *modularity FW* (*t*(59) = 2.382, *p_corr_* = 0.045) compared to the HC group. The HC group (*M* = 3.81, *SD* = 2.11, range = 2–8), D− group (*M* = 3.65, *SD* = 1.63, range = 2–9) and the D+ group (*M* = 3.33, *SD* = 1.23, range = 2–6) did not differ significantly on the number of modules detected (*χ^2^*(14) = 18.89, *p* = 0.17).

**Fig. 3:**
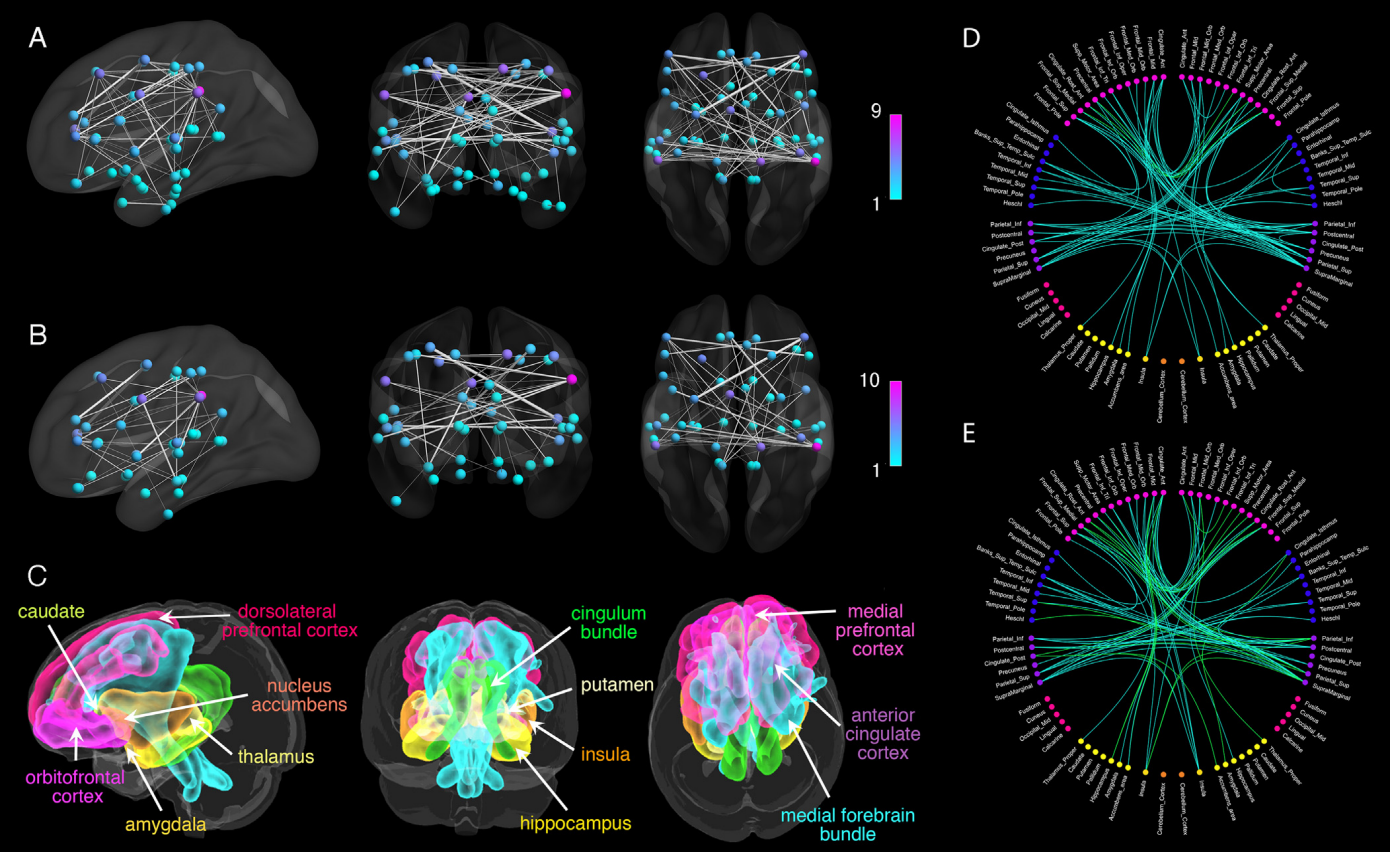
A) Network of significantly reduced fractional anisotropy (FA)-weighted connectivity in the group of stroke patients with depression compared to the healthy control group. B) Network of significantly increased free-water (FW)-weighted connectivity in the group of stroke patients with depression compared to the healthy control group. T-statistics are set to a supra-threshold of 3, which corresponds to *p* = 0.001. Subnetworks are significant at p_FWE_ < 0.05. Node colour reflects number of connections in subnetwork (degree of a node in the subnetwork), ranging from blue (few connections) to pink (many connections). Edge weights reflect t-statistic magnitude, whereby thicker edges correspond to higher t-statistics. C) Grey matter (warm colors) and white matter (cold colors) structures constituting the reward system. The medial forebrain bundle (cyan) and the cingulum bundle (green) interconnect the grey matter structures of the reward system. Connectograms of the significant D) FA and E) FW networks. Node color correspond to different lobes and subcortical regions: pink = frontal lobe, blue = temporal lobe, purple = parietal lobe, magenta = occipital lobe, yellow = subcortical structures, light orange = insular cortex, orange = cerebellum. Connection color represents F-statistics magnitude, ranging from cyan (low F-statistic value) to green (high F-statistic value). (For interpretation of the references to color in this figure legend, the reader is referred to the web version of this article.)

Compared to the HC group, the D+ group showed reduced FA-weighted connectivity (*p_FWE_* = 0.029) in a subnetwork comprising 47 nodes and 75 edges (see Fig. 3A). This network subsumed the reward system, in that it included 77% of nodes located in the reward system. Frontal-subcortical and within frontal lobe connections were a notable anatomical feature of this subnetwork. The majority (54%) of affected connections (edges) linked with frontal lobe nodes. The largest between group effect sizes were found in intrinsic connections within the frontal lobes, including between the medial superior frontal gyrus, precentral gyrus and rostral anterior cingulate gyrus. No connections showed significantly increased FA in the D− group compared to the HC group and no group differences in FA were observed between the D− group and the HC group or between D− and D+ groups.

A subnetwork of increased FW-weighted connectivity in the D+ group compared to the HC group (*p_FWE_* = 0.038) (see Fig. 3B) comprising 56 nodes and 79 edges was also demonstrated. Similar to the subnetwork based on FA differences, this subnetwork demonstrated an emphasis on connections (edges) to or within the frontal lobes (62%) and included 80% of nodes from the reward system. The largest between-group FW effect sizes were again located in intrinsic connections within the frontal lobes, including connections between the precentral gyrus, superior frontal gyrus, inferior frontal gyrus (pars triangularis) and rostral anterior cingulate cortex. No connections showed significantly decreased FW in the D+ group compared to the HC group and no group differences in FW were observed between the D− group and the HC group or the D− and D+ groups.

For a display of significant subnetworks across supra-thresholds of 3.5 (corresponding to *p* = 0.0005) and 4 (corresponding to *p* = 0.0001) see Supplementary Figs. S1 and S2.

### 3.3 Structural group differences in the reward system

A main effect of *group* was identified for *FA* (*F*(2,56) = 3.847, *p* = 0.033, *η_p_^2^* = 0.115). Polynomial contrasts of *group* found that FA significantly decreased linearly from the HC group, to the D− and the D+ group (*t*(59) = −2.264, *p* = 0.027). FA was significantly reduced in the left posterior cingulum subdivision in the D+ group (*t*(59) = 2.673, *p_corr_* = 0.029) and the D− group (*t*(59) = 3.09, *p_corr_* = 0.009) compared to the HC group. Significant group*tract(FW) (*F*(8,224) = 2.412, *p* = 0.016, *η_p_^2^* = 0.076) and group*tract(FW)*hemisphere (*F*(8,224) = 2.05, *p* = 0.042, *η_p_^2^* = 0.065) interactions were found, indicating that group differences in FW vary according to tract and hemisphere. FW was significantly increased in the right middle cingulum subdivision in the D− group compared to the HC group (*t*(59) = −3.039, *p_corr_* = 0.011) and in the left MFB in the D+ group compared to the HC group (*t*(59) = −2.594, *p_corr_* = 0.009) (see Fig. S3).

An ANCOVA performed in the stroke sample only, did not detect significant main effects for *vascular risk factors/vascular disease* or interactions with *region* or *hemisphere* (all *p* > 0.26), indicating no obvious association between vascular risk factors or vascular disease in stroke patients and changes in the reward system.

### 3.4 Contribution of global topology and measures in the reward system to depression severity

VIFs in the first regression analysis ranged from 1.01 to 1.52 before, and from 1.03 to 1.1 after removal of the outlier. VIFs in the second regression analysis ranged from 1.62 to 2.52 before, and from 1.18 to 2.54 after removal of the outlier. All VIFs were below the cutoff of 5, indicating that multicollinearity was not a problem in the models.

Global topology measures (together with the two covariates handedness and days since lesion) explained 21.7% (*R^2^_adj_* = 0.217, *F*(8,45) = 2.56, *p* = 0.025) of variance in GDS scores. *Modularity FW* was a significant independent predictor of GDS scores (*β* = 0.966, *partial r* = 0.269, *p* = 0.05) and *modularity FA* showed a non-significant trend for an independent effect (*β* = −0.866, *partial r* = −0.25, *p* = 0.069). When repeating the regression analysis without the participant who was a statistical outlier on GDS scores, the model did not remain significant (*R^2^_adj_* = 0.04, *F*(8,36) = 0.786, *p* = 0.618).

**Fig. 4:**
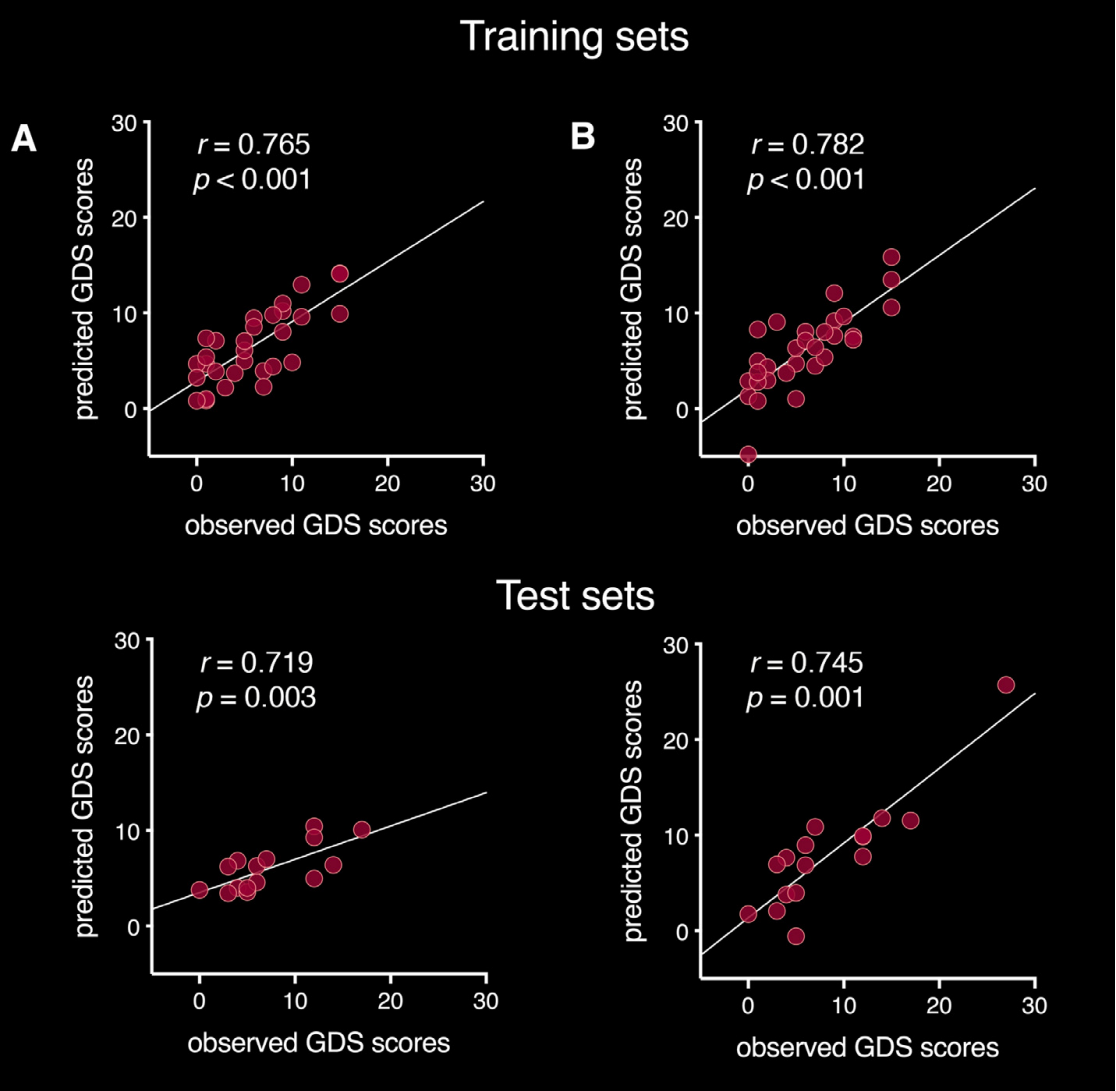
Correlations between Observed GDS scores plotted on the x-axis and predicted GDS scores from the regression models plotted on the y-axis. A) Correlations excluding the statistical outlier on GDS scores in the training set (upper scatterplot) and the test set (lower scatterplot). B) Correlations including the statistical outlier on GDS scores in the training set (upper scatterplot) and the test set (lower scatterplot).

The final model of the stepwise linear regression analysis with all measures of FA, FW and grey matter volume included 12 variables and explained 64.8% (*R^2^_adj_* = 0.648, *F*(14,18) = 6.912, *p* < 0.001) of the variance in GDS scores in the training set (70% participants). This model was used to estimate the predicted GDS scores in the test set (30% participants). Observed GDS scores correlated significantly with predicted GDS scores in the training set (*r* = 0.782, *p* < 0.001) and the test set (*r* = 0.745, *p* = 0.001; see Fig. 4). FA of the bilateral anterior and posterior, as well as the left parahippocampal cingulum subdivisions, FW in the MFB, right middle and left posterior cingulum subdivisions, as well as volumes of the left thalamus, bilateral OFC and right amygdala were significant independent predictors of GDS scores (see Table 2). When repeating the regression analysis without the statistical outlier on GDS scores, the final model included seven variables and explained 38.7% (*R^2^_adj_* = 0.387, *F*(9,23) = 4.089, *p* = 0.001) of the variance in GDS scores in the training set (70% participants). Predicted GDS scores from the test set were again estimated with this model. Predicted GDS scores in the training set (*r* = 0.765, *p* < 0.001) and the test set (*r* = 0.719, *p* = 0.003) correlated significantly with observed GDS scores (see Fig. 4). FA of the bilateral posterior and right middle cingulum subdivisions, FW in the left MFB, as well as left thalamus volume were significant independent predictors of GDS scores (see Table 2). The regression analysis remained significant after removal of the outlier but explained less (38.7% vs 64.8%) variance in GDS scores. This indicates that this data point influenced, but was not the sole driver of the results. Both regression models performed well in the test sets, which adds confidence that the models were not overfitted despite the large number of predictors.

**Table 2:** Regression analyses - predictors of GDS scores in final models.

	Training set^1^			Training set^2^		
predictor variable	β	t-statistic	p-value	β	t-statistic	p-value
*FA*						
AC_L_	0.44	2.93	0.006			
AC_R_	0.46	3.94	< 0.001			
MC_R_				0.73	4.67	< 0.001
PC_L_	−0.46	−3.69	0.001	−0.42	−3.27	0.002
PC_R_	−0.67	−5.22	< 0.001	−0.75	−4.71	< 0.001
PHC_L_	−0.26	−2.08	0.047	−0.23	−1.72	0.087

*FW*						
MC_R_	0.50	4.11	< 0.001	0.32	1.72	0.095
PC_L_	−0.34	−2.60	0.014			
MFB_L_	0.49	4.36	< 0.001	0.70	3.47	0.001

*GM*						
Th_L_	−0.43	−3.63	0.001	−0.41	−2.91	0.006
OFC_L_	0.63	3.00	0.005			
OFC_R_	−0.64	−3.80	0.009			
Amy_R_	0.25	2.10	0.044			

*Note.* GDS = geriatric depression scale; ^1^including outlier GDS = 27; ^2^excluding outlier GDS = 27; FA = fractional anisotropy; FW = free-water; GM = grey matter volume; L = left; R = right; AC = anterior cingulum subdivision; MC = middle cingulum subdivision; PC = posterior cingulum subdivision; PHC = parahippocampal cingulum; MFB = medial forebrain bundle; Th = thalamus; OFC = orbitofrontal cortex; Amy = amygdala.

## 4 Discussion

In this study, we found PSD to be associated with global network topology and subnetworks identified from structural connectome analysis that mapped predominantly onto fronto-subcortical regions and connections defined as constituting the reward system. Our focused analysis of grey and white matter correlates within the reward system showed that grey matter volumes of this circuit, together with FA and extracellular FW content of major connection pathways in this system were collectively predictive of PSD severity. These predominantly intrinsic frontal and fronto-subcortical subnetworks are typically disrupted in major depressive disorder (MDD). Our findings indicate that the structural basis of PSD, like MDD in the absence of stroke, resides in brain circuits typically involved in motivation, emotions and memory (Russo and Nestler, 2013). In PSD, these alterations may be remote from the infarct itself. Furthermore, to the extent that enhanced FW in the extracellular space may be indicative of inflammatory processes, our findings suggest that neuroinflammation may contribute to the development of PSD.

Graph theoretical analyses found that modularity estimated from FW was significantly increased in patients with PSD relative to healthy controls. Modularity indicates that nodes are forming communities via dense connections to one another, which have only sparse long-range connections to nodes from other modules. A recent study in individuals with treatment resistant depression found that noninvasive neurostimulation led to a significant reduction of depressive symptoms over time, which was associated with decreased modularity (Caeyenberghs et al., 2018). The authors concluded that transient changes in modular network configuration may be required to alleviate depressive symptoms. Increased FW could result from a number of processes that influence extracellular water content including neuroinflammation (Pasternak et al., 2012), atrophy (Ofori et al., 2015), neuronal depletion (Planetta et al., 2015) or axonal swelling (Pasternak et al., 2014). Our finding may suggest that clusters of pathological processes across the brain are linked to the development of PSD, possibly in conjunction with a simultaneous compromise of long-distance connections.

On closer examination of structures in the reward system, we observed structural changes in this circuit that account for much of the variability in depression after stroke. The origin of these observed changes, however, is unclear. Several explanations are plausible. 1.) It is conceivable that microstructural changes in the reward system represent an underlying liability for depression and that events such as a stroke, lead to the development of depressive symptoms. This is not the same as pre-existing depression. None of the participants reported pre-stroke mood symptoms. 2.) The observed changes in the reward system may be secondary to infarction elsewhere in the brain through neuronal degeneration distal to the lesion or neuroinflammatory processes along descending white matter pathways. 3.) Infarction and changes in the reward system may share a common causation, such as underlying vascular disease.

The grey matter volume changes and white matter alterations observed in our study closely resemble structural changes reported in MDD. Interestingly, family studies suggest that microstructural changes in the cingulum bundle may represent a biomarker of vulnerability for MDD (Keedwell et al., 2012). Furthermore, structural and functional abnormalities in the amygdala, nucleus accumbens, thalamus and orbitofrontal cortex (see Table S1) have been reported in MDD and also in healthy individuals with elevated levels of depressive symptoms (Webb et al., 2014) and familial risk of depression (Romanczuk-Seiferth et al., 2014). Taken together, these findings may suggest that individuals with altered cingulum bundle microstructure and grey matter changes in the reward system are already at increased risk for MDD and that the event of a stroke increases their chance of developing depressive symptoms (Webb et al., 2014). This might also explain why, similar to previous studies, we were unable to identify strong associations between lesion locations and PSD. Rather than being caused by injury to specific brain regions, it is possible that premorbid microstructural changes in the reward system may render some individuals more susceptible to develop PSD than others in the context of any given lesion.

Our global whole-brain connectome approach and the localized investigation of the reward system both identified increased FW volume fraction in fronto-subcortical connections in patients with PSD, but not in patients without PSD, relative to healthy controls. Freely diffusing water molecules are characteristic of cerebrospinal fluid and edema, but can also be indicative of subtler neuroinflammatory processes (Pasternak et al., 2012). This is because neuroinflammation increases the fractional volume of water molecules diffusing freely in the interstitial extracellular space, where microglia and other immunoreactive cells mediate immune defense (Pasternak et al., 2012). Neuroinflammation is induced via the release of pro-inflammatory cytokines in response to psychological stress, such as often preceding the onset of MDD, or physiological insult, as caused by stroke. Stroke elicits a cascade of neuroinflammatory events, which have neuroprotective properties and foster neuroplasticity, but can also cause secondary cell death (Ceulemans et al., 2010). Several regions in the reward system have been reported to be specifically vulnerable to neurotoxic effects of pro-inflammatory cytokines (Kim and Won, 2017), which, in turn, deplete serotonin and thereby contribute to the development of depressive symptoms (Fang and Cheng, 2009). Given that the patients in this study were scanned approximately 30–95 days post-stroke, it is conceivable that prolonged neuroinflammation in the reward system is a secondary consequence of lesions elsewhere in the brain.

Stroke is strongly associated with vascular risk factors such as diabetes mellitus, smoking, atrial fibrillation, ischemic heart disease, hypertension and cholesterol levels (Arboix, 2015). It is possible that vascular risk factors are a common causation of both, infarction and microstructural changes in the reward system. However, we did not observe any associations between vascular risk factors or vascular disease and structural measurements in the reward system, indicating that a common causation seems unlikely. While controlling for the effects of covariates in our analyses, only time since stroke was significantly associated with depressive symptoms, whereby depression severity increased with increasing number of days since stroke. This is particularly important as several neural connections regenerate and rearrange in the weeks to months after stroke. Reverse causality is also a possible explanation for this observation. For example, times to follow-up may have been longer in patients with more disability after stroke. Similarly, apathy, which partially overlaps with depression, may have led to longer follow-up intervals in some individuals. Following stroke survivors longitudinally and investigating disability, cognitive functioning and microstructural changes in the reward system in the acute phase compared to three to six months post-stroke, when depressive symptoms typically peak (Paolucci, 2008) would represent an important avenue for future research.

Estimates of white matter microstructure, grey matter volume and extracellular FW in the reward system were strongly associated with depression severity across the entire spectrum of GDS scores, indicating that its sensitivity is not limited to either high or low depression scores. Analysis without a participant with a very high GDS score confirmed that the measures collectively were predictive of GDS score, but suggested that the very high estimate for variance explained was a consequence of overfitting. This finding is particularly important as the characterization of highly sensitive, non-invasive neuroimaging biomarkers to identify individuals at high-risk for PSD is a necessary first step for the implementation of individualized interventions such as low dose antidepressants, anti-inflammatory medication and other prophylactic treatments to prevent the development of PSD. Interestingly, increasing evidence suggests that antidepressant medications possess anti-inflammatory properties (Hashioka, 2011), which may aid the recovery of structural damage restore serotonergic activity.

The present study had several limitations. Our finding of increased FA in the anterior middle cingulum might be biased by crossing projections in this area from the internal capsule. FA has previously been observed to be increased in the internal capsule (Coloigner et al., 2019) of patients with MDD. Future studies, using multi-shell diffusion MRI sequences may wish to investigate the crossing fiber populations in this region. Although the sample size was adequate to assess associations with sensitive and quantitative microstructural measures, it was insufficient for definitive voxel-wise analyses such as VLSM. However, previous VLSM studies (Gozzi et al., 2014, Kim et al., 2017, Terroni et al., 2011) and meta-analyses on lesion location (Narushima et al., 2003, Yu et al., 2004) have included large numbers of patients and have still been unable to identify brain regions specific to PSD. The purpose of our analysis of lesions was largely to show consistency with these studies and that there was no strong lesion effect arising as an idiosyncrasy of this sample. We used the Desikan-Killiany atlas for the reconstruction of the connectome and the extraction of grey matter volumes. While this atlas is widely used, it limits the parcellations to 84 regions. Better powered future studies may wish to investigate whether the findings observed in this study extend to different parcellation atlases with more regions. Lastly, in the absence of a stroke-free depression group, we were unable to determine whether the microstructural basis of post-stroke depression and major depressive disorder (MDD) overlap. Studying geriatric depression is confounded by the strong associations between diverse vascular pathologies and depressive symptoms, commonly referred to as vascular depression. This indicates that the etiology of depressive episodes may change over time. We suggest that prospective studies may benefit from a population-based design to investigate depression in individuals with and without incident vascular lesions, as well as investigate long-term exposures to risk states.

In summary, we found global and local structural brain changes to be associated with PSD. Specifically, PSD was strongly associated with white and grey matter measurements in the reward system, similar to those observed in MDD and independent of lesion location. Evidence of increased extracellular FW in the reward system might indicate a role for neuroinflammation in the development of PSD. Evaluation of structure within this system presents the opportunity to define biomarkers, which could identify individuals at high-risk for developing PSD, who might benefit from early interventions to prevent the development of depressive symptoms.

## Funding sources

This study was funded by the 10.13039/501100000265Medical Research Council, UK [grant reference MR/K022113/1] and the European Commission Horizon 2020 Health Programme [CoSTREAM, grant agreement 667375].

## Ethical standards

The authors assert that all procedures contributing to this work comply with the ethical standards of the relevant national and institutional committees on human experimentation and with the Helsinki Declaration of 1975, as revised in 2008.

## Conflicts of interest

Michael J. O’Sullivan has received support to attend meetings from Boehringer Ingelheim and received honoraria for consultancy from EMVison Medical Devices Ltd, Australia. The other authors have no conflicting interests to declare.

## CRediT authorship contribution statement

**Lena K.L. Oestreich:** Conceptualization, Methodology, Software, Formal analysis, Writing - original draft, Writing - review & editing, Visualization. **Paul Wright:** Validation, Investigation, Data curation. **Michael J. O’Sullivan:** Conceptualization, Resources, Writing - review & editing, Supervision, Project administration, Funding acquisition.
