# Supplementary material for: Microstructural changes in the reward system are associated with post-stroke depression
Source: Neuroimage Clin. 2020 Jul 22;28:102360. doi: 10.1016/j.nicl.2020.102360 (PMC7426585; doi:10.1016/j.nicl.2020.102360)
Supplement: Supplementary data 1 [file mmc1.docx]

1. Supplementary Methods

*Supplementary Table 1: Literature review of functional and structural MRI studies of the reward system in depression*

| *Table S1.* Literature review MRI studies of the reward system in depression | | |
| --- | --- | --- |
| brain region | reference | findings |
| ventral tegmental area (VTA) | Russo & Nestler (2013)  Geugies et al. (2019) | lower activity in patients with anhedonia (Geugies et al., 2019) |
| amygdala (Amy) | Russo & Nestler (2013)  Bora, Fornito, Pantelis, & Yucel (2012) | Reduced volume (Bora, Fornito, Pantelis, & Yucel, 2012; Russo & Nestler, 2013)  decreased BOLD activity during reward task (Russo & Nestler, 2013) |
| thalamus (Th) | Zhang et al (2013)  Mitterschiffthaler et al. (2003)  Segarra et al. (2016)  Ancelin et al. (2019) | reduced volume (Ancelin et al., 2019)  decreased BOLD activity during reward task (Mitterschiffthaler et al., 2003; Segarra et al., 2016; Zhang et al., 2013) |
| hippocampus (HPC) | Russo & Nestler (2013)  Campbell et al. (2004)  Geerlings & Gerritsen (2017)  Ballmaier et al. (2008) | Reduced volume (Ballmaier et al., 2008; Campbell, Marriott, Nahmias, & MacQueen, 2004; Geerlings & Gerritsen, 2017; Russo & Nestler, 2013)  decreased BOLD activity during reward task (Russo & Nestler, 2013) |
| nucleus accumbens (NAc) | Russo & Nestler (2013)  Pizzagalli et al. (2009)  Epstein et al. (2006)  Segarra et al. (2016)  Ancelin et al. (2019) | reduced volume (Ancelin et al., 2019; Russo & Nestler, 2013)  decreased BOLD activity at rest (Russo & Nestler, 2013) and during reward task (Epstein et al., 2006; Pizzagalli et al., 2009; Russo & Nestler, 2013; Segarra et al., 2016) |
| caudate (Cau) | Zhang et al (2013)  Enneking et al. (2019)  Keren et al. (2018)  Pizzagalli et al. (2009)  Epstein et al. (2006)  Forbes et al. (2009)  Segarra et al. (2016) | reduced volume in MDD with anhedonia (Keren et al., 2018; Pizzagalli et al., 2009) and increased symptom severity (Keren et al., 2018)  decreased BOLD activation during reward task (Epstein et al., 2006; Forbes et al., 2009; Keren et al., 2018; Pizzagalli et al., 2009; Segarra et al., 2016; Zhang et al., 2013) |
| putamen (Pu) | Zhang et al (2013)  Keren et al. (2019)  Mitterschiffthaler et al. (2003)  Robinson Cools, Carlisi, Sahakian, & Drevets. (2012)  Pizzagalli et al. (2009)  Epstein et al. (2006)  Klok, van Eijndhoven, Argyelan, Schene, & Tendolkar (2019) | reduced volume (Klok, van Eijndhoven, Argyelan, Schene, & Tendolkar, 2019)  decreased BOLD activity during reward tasks (Epstein et al., 2006; Keren et al., 2018; Mitterschiffthaler et al., 2003; Pizzagalli et al., 2009; Robinson, Cools, Carlisi, Sahakian, & Drevets, 2012; Zhang et al., 2013) |
| insula (Ins) | Zhang et al (2013)  Mitterschiffthaler et al. (2003)  Dichter et al. (2012)  Ancelin et al. (2019) | reduced volume (Ancelin et al., 2019)  decreased BOLD activity during reward task (Dichter, Kozink, McClernon, & Smoski, 2012; Mitterschiffthaler et al., 2003; Zhang et al., 2013) |
| dorsolateral prefrontal cortex (dlPFC) | Russo & Nestler (2013)  Forbes et al. (2009)  Bora et al. (2012) | reduced volume (Bora et al., 2012)  decreased BOLD activity during reward task (Russo & Nestler, 2013)  increased BOLD activity during reward anticipation (Forbes et al., 2009) |
| middle frontal gyrus (MFG) | Zhang et al (2013)  Dichter et al. (2012) | increased BOLD activity during reward anticipation (Dichter et al., 2012; Zhang et al., 2013) |
| superior frontal gyrus (SFG) | Zhang et al (2013) | increased BOLD activity during reward task (Zhang et al., 2013) |
| medial prefrontal cortex (mPFC) | Russo & Nestler (2013)  Mitterschiffthaler et al. (2003)  Epstein et al. (2006)  Forbes et al. (2009)  Segarra et al. (2016) | reduced volume (Russo & Nestler, 2013)  smaller BOLD activity during reward tasks (Epstein et al., 2006; Mitterschiffthaler et al., 2003; Russo & Nestler, 2013; Segarra et al., 2016)  increased BOLD activity during reward anticipation (Forbes et al., 2009) |
| orbitofrontal cortex (OFC) | Russo & Nestler (2013)  Mitterschiffthaler et al. (2003)  Dichter et al. (2012)  Segarra et al. (2016) | reduced volume (Russo & Nestler, 2013)  decreased BOLD activity during reward tasks (Dichter et al., 2012; Mitterschiffthaler et al., 2003; Segarra et al., 2016) |
| Medial orbitofron-tal cortex (mOFC) | Rothkirch, Tonn, Kohler, & Sterzer (2017) | decreased BOLD activity during reward task (Rothkirch, Tonn, Kohler, & Sterzer, 2017) |
| a­nterior cingulate cortex (ACC) | Russo & Nestler (2013)  Zhang et al (2013)  Mitterschiffthaler et al. (2003)  Dichter et al. (2012)  Bora et al. (2012) | reduced volume (Bora et al., 2012)  increased BOLD signal during reward task(Russo & Nestler, 2013) and reward anticipation (Dichter et al., 2012)  reduced BOLD signal during reward tasks (Mitterschiffthaler et al., 2003; Zhang et al., 2013) |

*Note.* MFG and SFG are part of dlPFC and mOFC is part of OFC.

*Tractography procedure*

Anatomical landmarks were identified on color-coded diffusion tensor maps. An exclusion region of interest (ROI) was drawn across the midline sagittal plane to exclude interhemispheric projections. Further exclusion ROIs were drawn to exclude tracts that deviated from the anatomy of the cingulum bundle or medial forebrain bundle. All cingulum subdivisions and the medial forebrain bundle were reconstructed in the left and right hemisphere.

The anterior, middle, posterior and parahippocampal subdivisions were reconstructed as described by Metzler-Baddeley et al (2012), with minor modifications (Metzler-Baddeley et al., 2012): The anterior cingulum was defined as the cingulum segment rostral to the anterior commissure. The seed ROI was drawn in line with the anterior commissure in the coronal plane. One inclusion ROI was placed in the slice where the most inferior part of the genu can be identified in the axial plane and another inclusion ROI was drawn in the coronal plane where the most posterior part of the genu is visible. The middle cingulum was defined as the cingulum segment located between the anterior and posterior commissure. The seed ROI was drawn in line with the anterior commissure and one inclusion ROI was drawn in line with the posterior commissure in the coronal plane. The posterior cingulum was defined as the cingulum segment caudal to the posterior commissure. The seed ROI was placed in line with the posterior commissure in the coronal plane. One inclusion ROI was drawn in the slice where the most inferior part of the splenium can be identified in the axial plane and another inclusion ROI was placed in the coronal plane where the most anterior part of the splenium is visible. The parahippocampal cingulum was reconstructed by placing a seed ROI in the coronal plane where the most posterior part of the splenium is visible. One inclusion ROI was drawn below the splenium in the most anterior slice where the anterior splenium can be seen. The medial forebrain bundle was reconstructed based on adapted protocols by Coenen et al. (2012) (Coenen, Panksepp, Hurwitz, Urbach, & Madler, 2012) and Anthofer et al. (2015) (Anthofer et al., 2015): A seed ROI was placed in the medial part of the ventral tegmental area in the axial plane. One inclusion ROI was drawn in the axial plane at the junction of the caudate nucleus and nucleus accumbens.

1. Supplementary Results

*Whole-brain topology and connectome analysis*


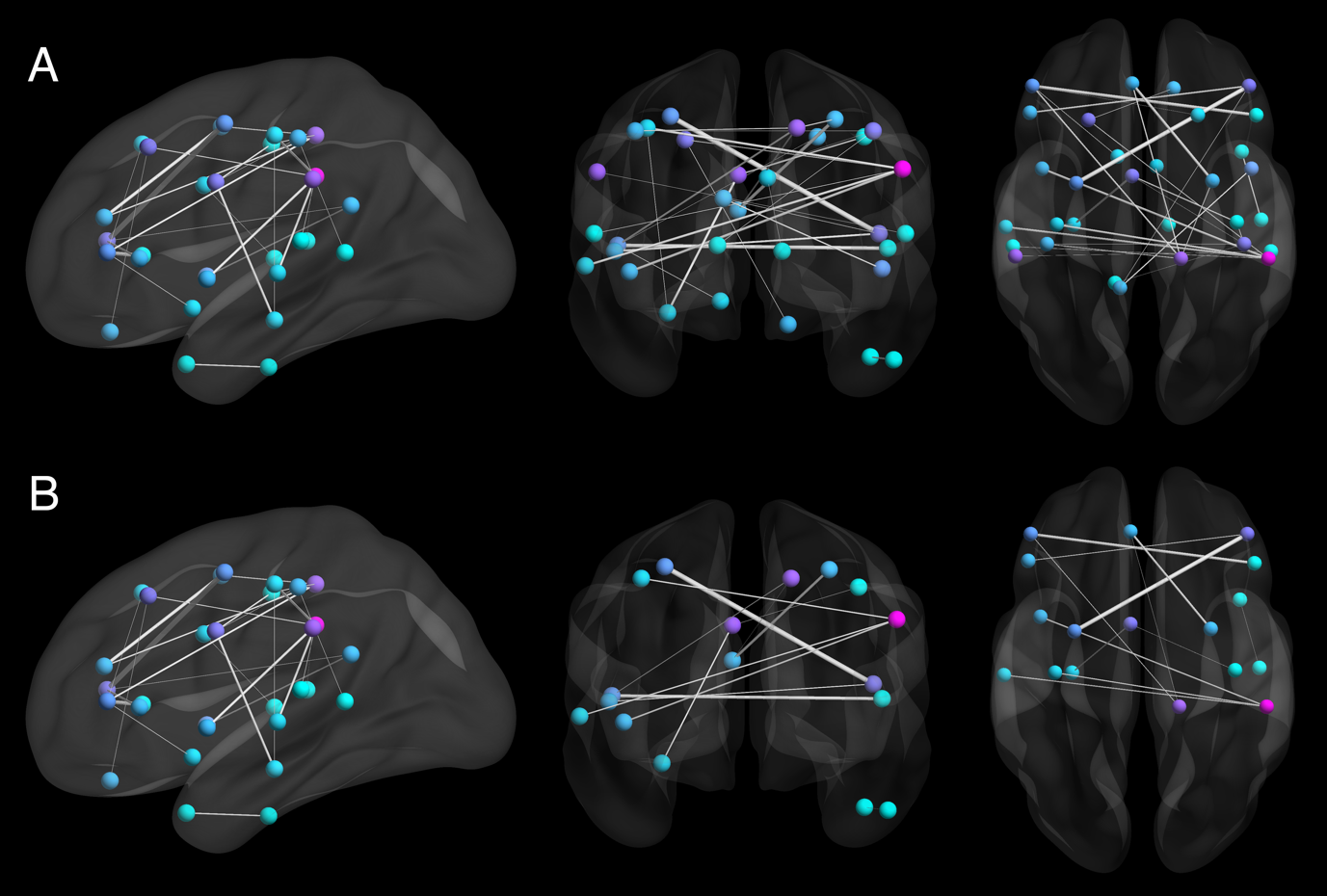


*Figure S1.* Networks of significantly reduced FA in the group of patients with post-stroke depression compared to the healthy control group. Node colour reflects number of connections in subnetwork (degree of a node in the subnetwork), ranging from blue (few connections) to pink (many connections). Edge weights reflect t-statistic magnitude, whereby thicker edges correspond to higher t-statistics. Subnetworks are significant at *p_FWE_* < 0.05. A) T-statistic is set to a supra-threshold of 3.5, which corresponds to *p* = 0.0005. B) T-statistic is set to a supra-threshold of 4, which corresponds to *p* = 0.0001.


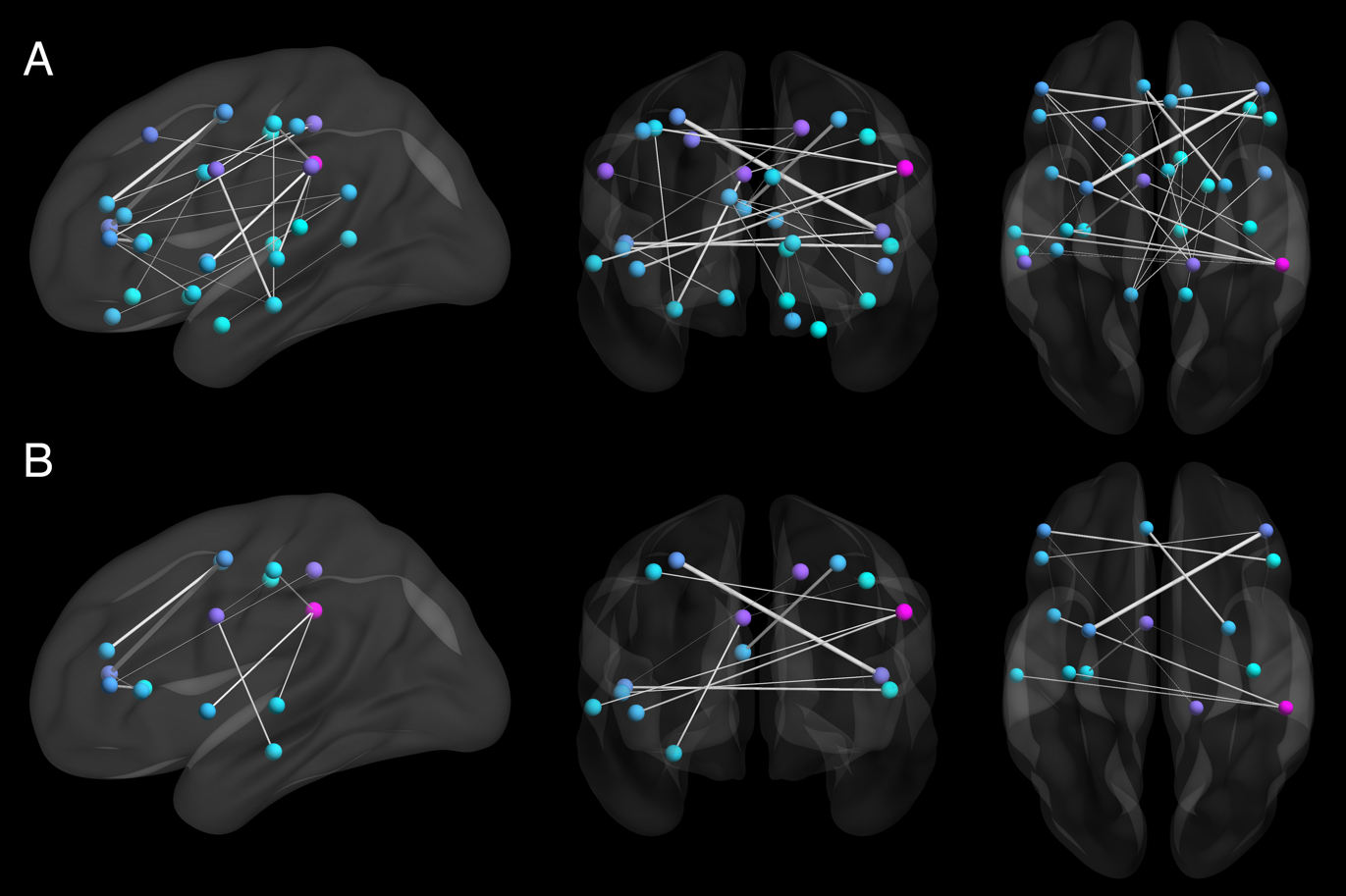


*Figure S2.* Networks of significantly reduced free-water in the group of patients with post-stroke depression compared to the healthy control group. Node colour reflects number of connections in subnetwork (degree of a node in the subnetwork), ranging from blue (few connections) to pink (many connections). Edge weights reflect t-statistic magnitude, whereby thicker edges correspond to higher t-statistics. Subnetworks are significant at *p_FWE_* < 0.05. A) T-statistic is set to a supra-threshold of 3.5, which corresponds to *p* = 0.0005 B) T-statistic is set to a supra-threshold of 4, which corresponds to *p* = 0.0001.

- 1. *Structural group differences in the reward system*

Compared to the HC group, the D+ group had significantly reduced FA across regions and hemispheres (*t*(59) = 2.4, *p_corr_* = 0.045) and the D- group showed a trend towards reduced overall FA (*t*(59) = 2.375, *p_corr_* = 0.053) (see Figure S4).


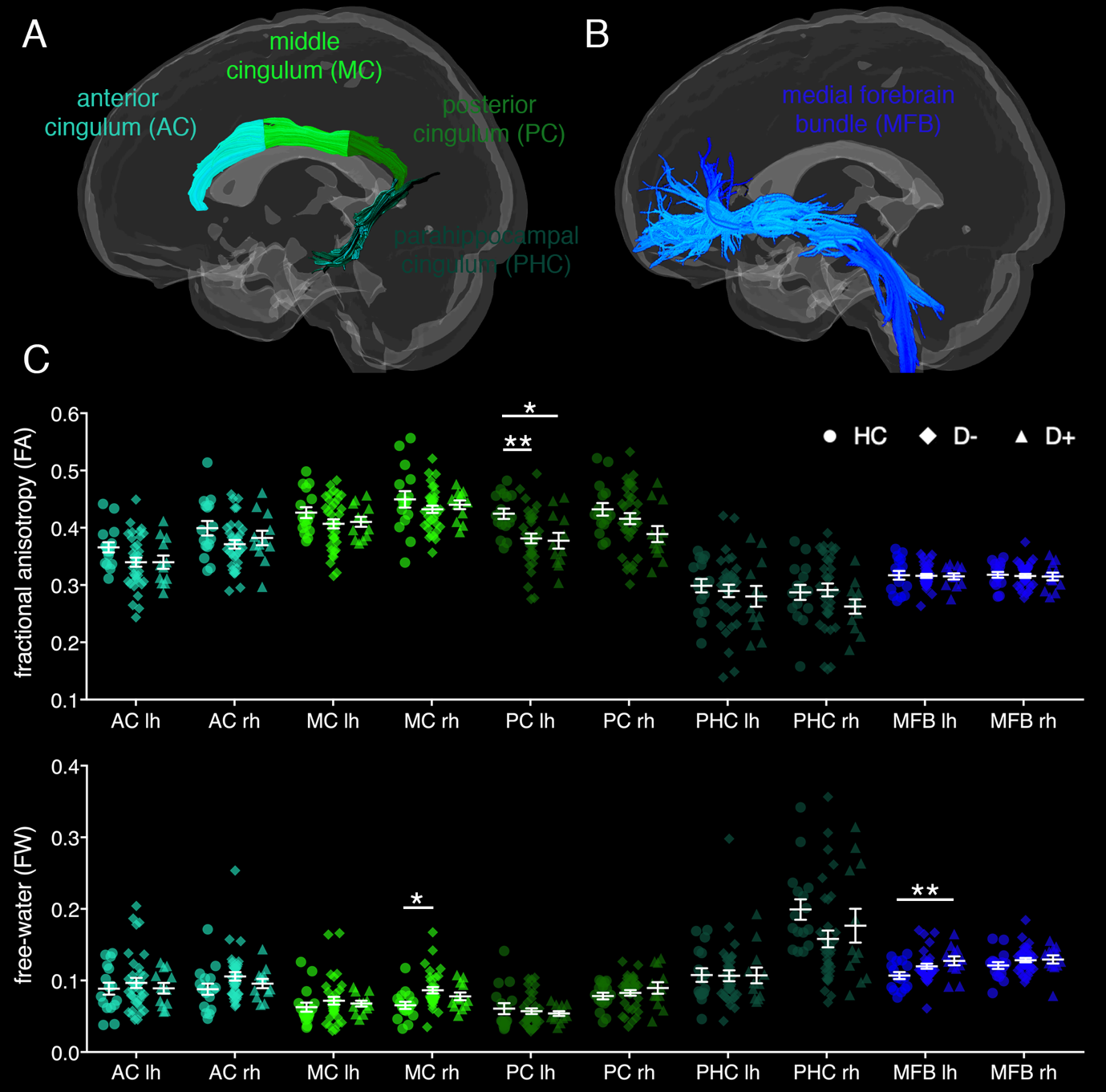


*Figure S3.* A) Cingulum bundle subdivisions from one representative participant B) Medial forebrain bundle (MFB) reconstruction from one representative participant C) fractional anisotropy (FA) and free-water (FW) values displayed separately for the healthy control group, as well as the groups of stroke patients without depression (D-) and with depression (D+). *Bonferroni corrected significant (*p* < 0.05) difference between groups **Bonferroni corrected significant (*p* < 0.05) difference between groups.

A repeated-measures ANCOVA with grey matter volumes did not detect a main effect for group or any significant interactions with group (see Figure S5).


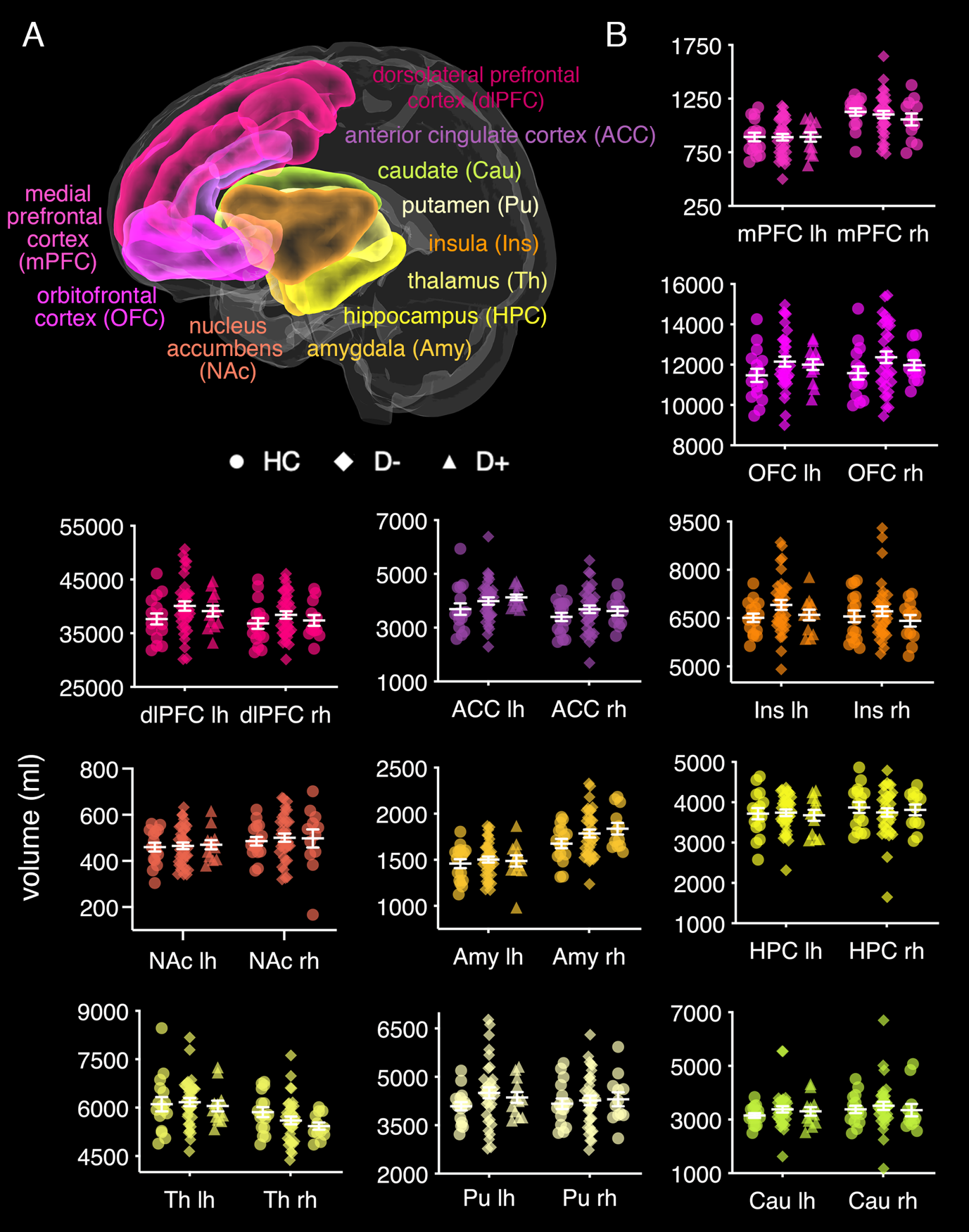


*Figure S4*. C) Grey matter structures of the reward system and B) grey matter volume (ml) displayed separately for the healthy control group, as well as the groups of stroke patients without depression (D-) and with depression (D+).

References

Ancelin, M. L., Carriere, I., Artero, S., Maller, J., Meslin, C., Ritchie, K., . . . Chaudieu, I. (2019). Lifetime major depression and grey-matter volume. *Journal of Psychiatry and Neuroscience, 44*(1), 45-53. doi:10.1503/jpn.180026

Anthofer, J. M., Steib, K., Fellner, C., Lange, M., Brawanski, A., & Schlaier, J. (2015). DTI-based deterministic fibre tracking of the medial forebrain bundle. *Acta Neurochirurgica, 157*(3), 469-477. doi:10.1007/s00701-014-2335-y

Ballmaier, M., Narr, K. L., Toga, A. W., Elderkin-Thompson, V., Thompson, P. M., Hamilton, L., . . . Kumar, A. (2008). Hippocampal morphology and distinguishing late-onset from early-onset elderly depression. *American Journal of Psychiatry, 165*(2), 229-237. doi:10.1176/appi.ajp.2007.07030506

Bora, E., Fornito, A., Pantelis, C., & Yucel, M. (2012). Gray matter abnormalities in Major Depressive Disorder: a meta-analysis of voxel based morphometry studies. *Journal of Affective Disorders, 138*(1-2), 9-18. doi:10.1016/j.jad.2011.03.049

Campbell, S., Marriott, M., Nahmias, C., & MacQueen, G. M. (2004). Lower hippocampal volume in patients suffering from depression: a meta-analysis. *American Journal of Psychiatry, 161*(4), 598-607. doi:10.1176/appi.ajp.161.4.598

Coenen, V. A., Panksepp, J., Hurwitz, T. A., Urbach, H., & Madler, B. (2012). Human medial forebrain bundle (MFB) and anterior thalamic radiation (ATR): imaging of two major subcortical pathways and the dynamic balance of opposite affects in understanding depression. *Journal of Neuropsychiatry and Clinical Neurosciences, 24*(2), 223-236. doi:10.1176/appi.neuropsych.11080180

Corp., I. (2017). IBM SPSS Statistics for Macintosh (Version 25.0). Armonk, NY: IBM Corp.

Craney, T. A., & Surles, J. G. (2002). Model-Dependent Variance Inflation Factor Cutoff Values. *Quality Engineering, 14*(3), 391-403. doi:10.1081/QEN-120001878

Dale, A. M., Fischl, B., & Sereno, M. I. (1999). Cortical surface-based analysis. I. Segmentation and surface reconstruction. *Neuroimage, 9*(2), 179-194. doi:10.1006/nimg.1998.0395

Dichter, G. S., Kozink, R. V., McClernon, F. J., & Smoski, M. J. (2012). Remitted major depression is characterized by reward network hyperactivation during reward anticipation and hypoactivation during reward outcomes. *Journal of Affective Disorders, 136*(3), 1126-1134. doi:10.1016/j.jad.2011.09.048

Epstein, J., Pan, H., Kocsis, J. H., Yang, Y., Butler, T., Chusid, J., . . . Silbersweig, D. A. (2006). Lack of ventral striatal response to positive stimuli in depressed versus normal subjects. *American Journal of Psychiatry, 163*(10), 1784-1790. doi:10.1176/ajp.2006.163.10.1784

Forbes, E. E., Hariri, A. R., Martin, S. L., Silk, J. S., Moyles, D. L., Fisher, P. M., . . . Dahl, R. E. (2009). Altered striatal activation predicting real-world positive affect in adolescent major depressive disorder. *American Journal of Psychiatry, 166*(1), 64-73. doi:10.1176/appi.ajp.2008.07081336

Geerlings, M. I., & Gerritsen, L. (2017). Late-Life Depression, Hippocampal Volumes, and Hypothalamic-Pituitary-Adrenal Axis Regulation: A Systematic Review and Meta-analysis. *Biological Psychiatry, 82*(5), 339-350. doi:10.1016/j.biopsych.2016.12.032

Geugies, H., Mocking, R. J. T., Figueroa, C. A., Groot, P. F. C., Marsman, J. C., Servaas, M. N., . . . Ruhe, H. G. (2019). Impaired reward-related learning signals in remitted unmedicated patients with recurrent depression. *Brain, 142*(8), 2510-2522. doi:10.1093/brain/awz167

Keren, H., O'Callaghan, G., Vidal-Ribas, P., Buzzell, G. A., Brotman, M. A., Leibenluft, E., . . . Stringaris, A. (2018). Reward Processing in Depression: A Conceptual and Meta-Analytic Review Across fMRI and EEG Studies. *The American journal of psychiatry, 175*(11), 1111-1120. doi:10.1176/appi.ajp.2018.17101124

Klok, M. P. C., van Eijndhoven, P. F., Argyelan, M., Schene, A. H., & Tendolkar, I. (2019). Structural brain characteristics in treatment-resistant depression: review of magnetic resonance imaging studies. *BJPsych Open, 5*(5), e76. doi:10.1192/bjo.2019.58

Metzler-Baddeley, C., Jones, D. K., Steventon, J., Westacott, L., Aggleton, J. P., & O'Sullivan, M. J. (2012). Cingulum Microstructure Predicts Cognitive Control in Older Age and Mild Cognitive Impairment. *The Journal of Neuroscience, 32*(49), 17612-17619. doi:10.1523/jneurosci.3299-12.2012

Mitterschiffthaler, M. T., Kumari, V., Malhi, G. S., Brown, R. G., Giampietro, V. P., Brammer, M. J., . . . Sharma, T. (2003). Neural response to pleasant stimuli in anhedonia: an fMRI study. *Neuroreport, 14*(2), 177-182. doi:10.1097/00001756-200302100-00003

Pasternak, O., Sochen, N., Gur, Y., Intrator, N., & Assaf, Y. (2009). Free water elimination and mapping from diffusion MRI. *Magnetic Resonance in Medicine, 62*(3), 717-730. doi:10.1002/mrm.22055

Pizzagalli, D. A., Holmes, A. J., Dillon, D. G., Goetz, E. L., Birk, J. L., Bogdan, R., . . . Fava, M. (2009). Reduced caudate and nucleus accumbens response to rewards in unmedicated individuals with major depressive disorder. *American Journal of Psychiatry, 166*(6), 702-710. doi:10.1176/appi.ajp.2008.08081201

Robinson, O. J., Cools, R., Carlisi, C. O., Sahakian, B. J., & Drevets, W. C. (2012). Ventral striatum response during reward and punishment reversal learning in unmedicated major depressive disorder. *The American journal of psychiatry, 169*(2), 152-159. doi:10.1176/appi.ajp.2011.11010137

Rothkirch, M., Tonn, J., Kohler, S., & Sterzer, P. (2017). Neural mechanisms of reinforcement learning in unmedicated patients with major depressive disorder. *Brain, 140*(4), 1147-1157. doi:10.1093/brain/awx025

Russo, S. J., & Nestler, E. J. (2013). The brain reward circuitry in mood disorders. *Nature Reviews Neuroscience, 14*, 609. doi:10.1038/nrn3381

Segarra, N., Metastasio, A., Ziauddeen, H., Spencer, J., Reinders, N. R., Dudas, R. B., . . . Murray, G. K. (2016). Abnormal Frontostriatal Activity During Unexpected Reward Receipt in Depression and Schizophrenia: Relationship to Anhedonia. *Neuropsychopharmacology : official publication of the American College of Neuropsychopharmacology, 41*(8), 2001-2010. doi:10.1038/npp.2015.370

Smith, R. E., Tournier, J.-D., Calamante, F., & Connelly, A. (2012). Anatomically-constrained tractography: Improved diffusion MRI streamlines tractography through effective use of anatomical information. *Neuroimage, 62*(3), 1924-1938. doi:<http://dx.doi.org/10.1016/j.neuroimage.2012.06.005>

Smith, R. E., Tournier, J. D., Calamante, F., & Connelly, A. (2013). SIFT: Spherical-deconvolution informed filtering of tractograms. *Neuroimage, 67*, 298-312. doi:10.1016/j.neuroimage.2012.11.049

Tournier, J. D., Calamante, F., & Connelly, A. (2012). MRtrix: Diffusion tractography in crossing fiber regions. *International Journal of Imaging Systems and Technology, 22*(1), 53-66. doi:10.1002/ima.22005

Tournier, J. D., Calamante, F., & Connelly, A. (2013). Determination of the appropriate b value and number of gradient directions for high-angular-resolution diffusion-weighted imaging. *NMR in Biomedicine, 26*(12), 1775-1786. doi:10.1002/nbm.3017

Zalesky, A., Fornito, A., & Bullmore, E. T. (2010). Network-based statistic: Identifying differences in brain networks. *Neuroimage, 53*(4), 1197-1207. doi:10.1016/j.neuroimage.2010.06.041

Zhang, W.-N., Chang, S.-H., Guo, L.-Y., Zhang, K.-L., & Wang, J. (2013). The neural correlates of reward-related processing in major depressive disorder: A meta-analysis of functional magnetic resonance imaging studies. *Journal of Affective Disorders, 151*(2), 531-539. doi:10.1016/j.jad.2013.06.039
